# Identification and validation of icaritin-associated prognostic genes in hepatocellular carcinoma through network pharmacology, bioinformatics analysis, and cellular experiments

**DOI:** 10.3389/fimmu.2025.1693028

**Published:** 2025-11-20

**Authors:** Yaru Shi, Yanan Bai, Jianglan Wu, Yunfeng Yu, Xinyu Yang, Haobo Yang, Weixiong Jian, Jun Qing

**Affiliations:** 1 School of Traditional Chinese Medicine, Hunan University of Traditional Chinese Medicine, Changsha, Hunan, China; 2 Diagnostics of Traditional Chinese Medicine, National Key Discipline, Hunan University of Traditional Chinese Medicine, Changsha, Hunan, China; 3 Department of Cardiovascular Medicine, Hunan Hospital of Integrated Traditional Chinese and Western Medicine, Changsha, Hunan, China

**Keywords:** hepatocellular carcinoma, icaritin, prognostic genes, molecular mechanism, network pharmacology, bioinformatics analysis

## Abstract

**Background:**

Hepatocellular carcinoma (HCC) is a key global health issue, marked by poor clinical outcomes and lower survival rates. Icaritin (ICT), a bioactive compound derived from traditional Chinese medicine, has shown promising multi-target antitumor properties and potential clinical benefits in the treatment of HCC; however, its precise mechanisms of action remain insufficiently understood. Therefore, this study adopted an integrative strategy that combined bioinformatics analysis, experimental validation, and network pharmacology to systematically explore the prognostic and therapeutic relevance of ICT-associated genes.

**Methods:**

Initially, potential targets of ICT and HCC-associated genes were identified through extensive database screening, and the overlapping candidates were further determined using WGCNA and differential expression analysis. These core intersecting genes were subsequently refined via four complementary machine learning algorithms, KM survival analysis and LASSO Cox regression to establish a prognostic risk score model with predictive value. Additionally, molecular docking and dynamics simulations were performed to evaluate the binding stability between ICT and these targets. Finally, *in vitro* experiments were conducted to evaluate the effects of ICT on the proliferation and migration, as well as the expression of core target genes.

**Results:**

We identified thirty-five overlapping targets between ICT and HCC, and functional enrichment analysis showed that these genes are primarily implicated in cell cycle regulation and glycolytic pathways, highlighting potential mechanisms through which ICT exerts its antitumor effects. By integrating multiple machine learning approaches, KM survival analysis and LASSO Cox regression, we developed a four-gene prognostic model that successfully stratified HCC patients into higher- and lower-risk groups. Molecular docking and molecular dynamics simulations demonstrated that ICT binds stably to core targets, supporting its potential role in modulating disease progression. *In vitro* validation confirmed that ICT suppresses HepG2 and Huh7 cells proliferation and migration in a dose-dependent manner, while molecular analyses demonstrated that ICT treatment significantly downregulates CA9, UCK2, and FABP5 expression and simultaneously upregulates CYP2C9, thereby supporting its role in modulating critical oncogenic pathways.

**Conclusion:**

Modulation of ICT-targeted genes was found to effectively suppress HCC progression, underscoring their potential value as prognostic biomarkers and ideal therapeutic targets for the treatment of HCC.

## 1 Introduction

Liver cancer is the 6th most prevalent malignant neoplasm around the globe and the second key cause of cancer-related mortality (1). Among its subtypes, hepatocellular carcinoma (HCC) accounts for around 80–90% of primary cases of liver cancer and is characterized by an unfavorable prognosis, with a 5-year survival rate as low as 12%, thereby posing a substantial global public health burden (2, 3). China bears nearly half of the global liver cancer cases, reflecting a disproportionate disease burden (4). The major etiological factors contributing to HCC include chronic liver disorders such as viral hepatitis, liver cirrhosis, and excessive alcohol consumption. Surgical resection remains the primary therapeutic option; however, because early-stage HCC is frequently asymptomatic or associated with nonspecific clinical manifestations, fewer than 30% of patients are qualified for curative surgery at diagnosis, leaving most cases detected at advanced stages when surgical intervention is no longer feasible (5). For advanced HCC, systemic therapies such as lenvatinib and sorafenib are recommended, but their clinical efficacy is often compromised by treatment resistance, disease progression, and prolonged drug-related toxicities, which collectively limit therapeutic outcomes (6). Moreover, even after apparently successful surgical resection, many patients face a higher risk of recurrence or metastasis, contributing to persistently poor long-term survival rates (7). These challenges underscore an urgent necessity for the advancement of safer and more effective therapeutic strategies to advance survival outcomes and enhance the quality of life (QoL) for HCC patients.

Growing evidence indicates that traditional Chinese medicine (TCM) and natural compounds hold systemic therapeutic potential in managing HCC, as they have been shown to reduce treatment-related toxicity, enhance therapeutic efficacy, suppress tumor recurrence, improve QoL, and prolong overall survival (OS) (8–10). Among these agents, Icaritin (ICT), a prenylated flavonoid isolated from the medicinal herb Epimedium, has demonstrated potent anti-tumor activity in HCC as well as in several other malignancies (11, 12). The anti-cancer effects of ICT are mediated via the multiple signaling pathways’ regulation, including ERK/ULK1/NCOA4, IL-6/JAK2/STAT3, and ER-α36 (13, 14). Clinical trials involving advanced HCC patients, particularly those unable to tolerate conventional therapies and generally facing poor prognoses, have further shown that ICT is well tolerated with minimal adverse events, while also improving survival outcomes, delaying disease progression, and delivering meaningful clinical benefits (15). In addition, ICT has been reported to boost anti-tumor immune responses by modulating the phenotype and function of key immune cells, such as MDSCs and CD8+ T cells (16). Together, these results underscore ICT’s promise as a novel therapeutic candidate for HCC and other cancers; nevertheless, the accurate molecular mechanisms underlying its anti-HCC effects continue to be fully explained.

Network pharmacology, as an integrative multidisciplinary methodology, addresses the inherent limitations of traditional single-target research by constructing comprehensive “drug–target–pathway–disease” networks that systematically analyze complex biological interactions, thereby reflecting the multi-target synergistic effects characteristic of TCM (17). This systems-level approach offers a novel framework for elucidating the intricate gene networks and biological processes (BPs) underlying ICT’s therapeutic effects in HCC, offering insights into its multi-target mechanisms and potential contributions to improved treatment outcomes. Complementing this strategy, molecular docking serves as a widely applied computational technique that simulates the interactions between small molecules (ligands) and proteins (receptors) by predicting binding conformations and estimating affinity parameters (18). Its higher predictive accuracy and cost-effectiveness have made molecular docking be a vital tool in structural biology, modern drug discovery, and the exploration of biochemical pathways. In parallel, bioinformatics plays a critical role across nearly all phases of drug development, extending far beyond the processing of large-scale datasets to provide powerful predictive, analytical, and interpretive capabilities that inform both preclinical research and clinical applications.

**Figure 1:**
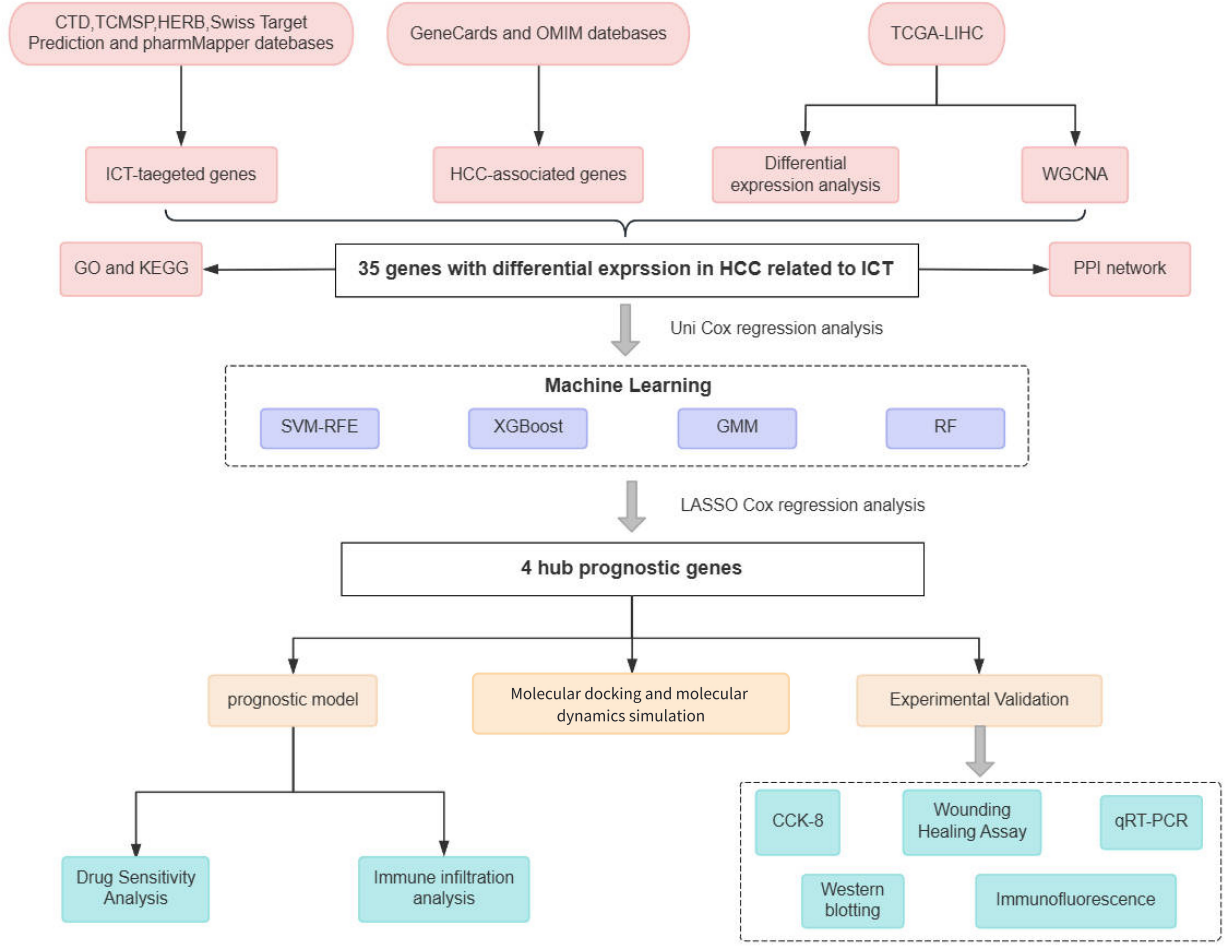
Flowchart of this study.

This study applied an integrative approach that combined network pharmacology and bioinformatics analyses to identify core genes targeted by ICT in HCC and to evaluate their prognostic significance. Based on ICT-related genes closely associated with survival outcomes, we constructed a prognostic model and further explored the relationship between gene expression levels and pharmacological sensitivity. Molecular docking was then performed to predict the binding affinities of ICT with its potential target proteins. To substantiate these computational findings, experimental validation was carried out using human hepatoma HepG2 cell lines, confirming the inhibitory effects of ICT on cell proliferation, migration, and target gene expression. By integrating advanced computational methodologies with *in vitro* experiments, this investigation not only elucidates the prognostic and therapeutic relevance of ICT-related genes in HCC but also provides novel insights and a theoretical foundation for the development of effective treatment strategies utilizing small-molecule compounds derived from TCM. The flowchart of this study is shown in **Figure 1**.

## 2 Materials and methods

### 2.1 Network pharmacology and bioinformatics

#### 2.1.1 Identify potential targets for ICT and HCC

**Figure 2:**
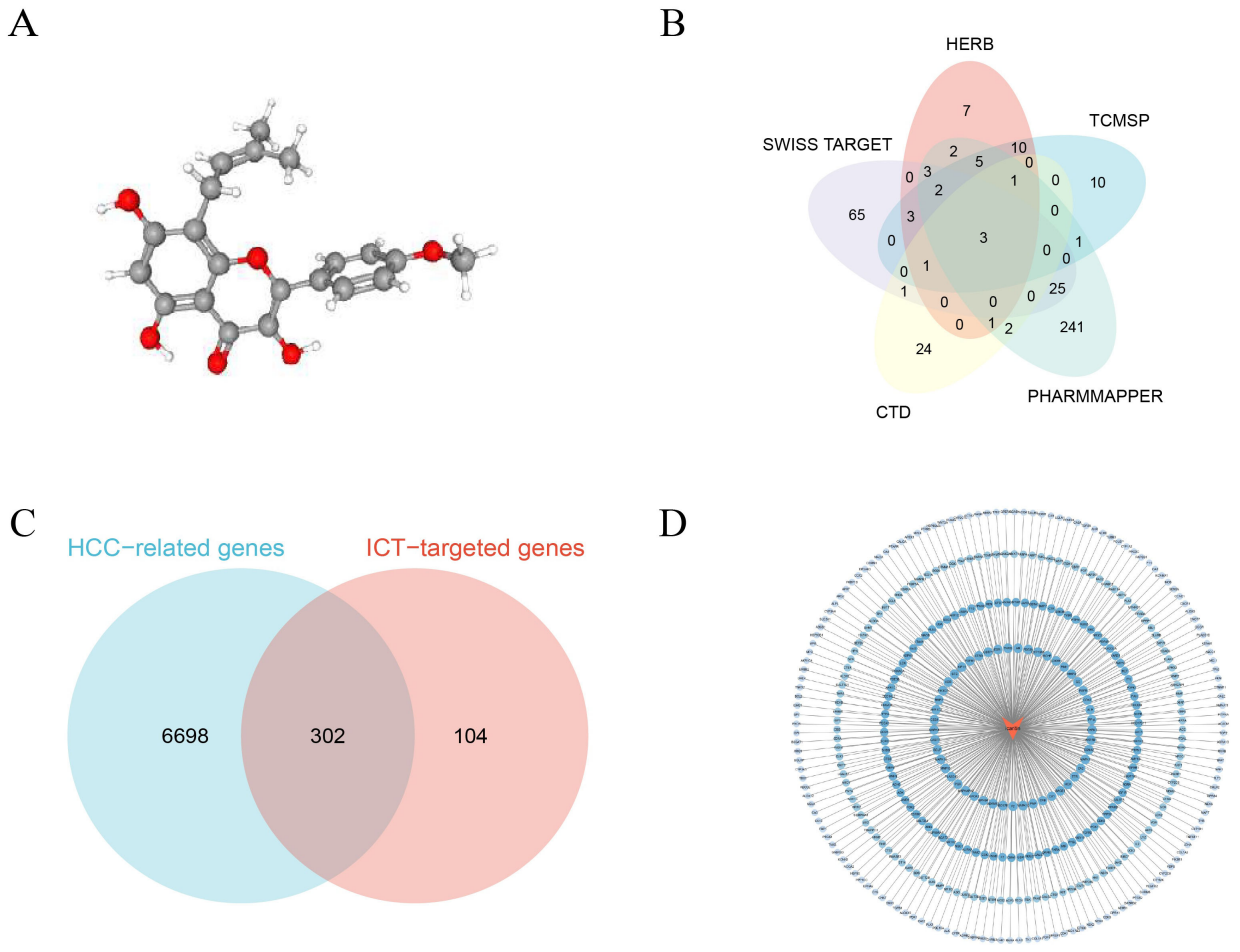
Network pharmacology analysis of ICT. **(A)** Three-dimensional chemical structure of ICT (PubChem CID: 5318980 with the molecular formula C_21_H_20_O_6_). **(B)** Candidate therapeutic targets of ICT retrieved from five public databases. **(C)** Venn diagram illustrating the overlapping genes between potential ICT targets and HCC-associated genes. **(D)** PPI network of ICT targets.

The ADME properties of ICT were retrieved from the TCMSP (https://tcmsp-e.com/) database (19), revealing an oral bioavailability (OB) of 45.41% and a drug-likeness (DL) index of 0.44. OB reflects the rate and extent to which a compound enters systemic circulation, serving as a key determinant of therapeutic efficacy, while DL indicates the likelihood that a compound possesses favorable pharmacokinetic properties based on its functional groups and physicochemical characteristics. According to TCMSP criteria, compounds with OB≥30% and DL≥0.18 are considered active candidates, suggesting that ICT likely exhibits significant pharmacological activity *in vivo*. The 3D molecular structure of ICT was sourced from the PubChem (http://pubchem.ncbi.nlm.nih.gov/) database (20), as shown in **Figure 2A**.

Potential ICT-related target genes were recognized by systematically searching three public databases, CTD (http://ctdbase.org/) (21), TCMSP, and HERB (http://herb.ac.cn/) (22), using the keyword “icaritin.” To further expand target prediction, both the SMILES format and three-dimensional (3D) molecular structure of ICT were obtained from the PubChem database. The SMILES format was subsequently entered into the Swiss Target Prediction platform (http://www.swisstargetprediction.ch/) (23) to forecast potential molecular targets, while the 3D structure was uploaded to the PharmMapper database (https://www.lilab-ecust.cn/pharmmapper/index.html) (24), where gene targets with a normalized fitting score (Normfit) greater than 0.3 were selected. Screening “ICT” across five databases, CTD, TCMSP, HERB, Swiss Target Prediction, and PharmMapper, identified 33, 36, 38, 103, and 289 potential targets, respectively; after integration and removal of duplicates, 406 unique ICT-related targets were compiled (**Figure 2B**).

For the identification of HCC-related genes, the GeneCards (https://www.genecards.org) and OMIM (https://www.omim.org) databases were systematically queried using the keywords “hepatocellular carcinoma” and “HCC”. Within the GeneCards database, only genes with a relevance score ≥ 2.0 were selected as higher-confidence candidates. The gene sets obtained from both databases were subsequently integrated, and duplicate entries were deleted to produce a non-redundant and comprehensive list of HCC-associated targets. The OMIM and GeneCards databases yielded 192 and 6,874 entries, respectively, which, after deduplication, resulted in 7,000 distinct HCC-associated genes. The overlapping targets between ICT-related and HCC-associated genes are presented in a Venn diagram (**Figure 2C**), and the ICT compound–target interaction network was then constructed using Cytoscape 3.10.2 (**Figure 2D**).

#### 2.1.2 Differential expression analysis and co-expression network construction

Transcriptomic sequencing data and relevant clinical information for HCC samples were obtained from the TCGA-LIHC cohort in the TCGA database (https://portal.gdc.cancer.gov/), which comprises 374 tumor samples and 50 adjacent normal controls. DEA was performed on count-based gene expression data using the “DESeq2” package in R software (version 4.3.3), with genes classified as differentially expressed genes (DEGs) when adjusted *p*-values were < 0.05 and |log2 fold change (FC)| ≥ 1. The results were imaged utilizing the “ggplot2” package to generate a volcano plot of DEGs. For downstream analyses, HCC expression profiles were normalized to transcripts per million (TPM). Weighted gene co-expression network analysis (WGCNA) was then employed to identify gene modules functionally associated with HCC, with the top 25% of genes showing the highest variance selected as input. Prior to network construction, outlier samples were identified and excluded using the “goodSamplesGenes” function, and data consistency was assessed through sample clustering. The optimum soft-thresholding power was determined utilizing the “pickSoftThreshold” function, enabling conversion of the correlation matrix into an adjacency matrix and subsequent construction of a topological overlap matrix (TOM). Moreover, gene modules were observed via hierarchical clustering, with highly similar modules merged at a mergeCutHeight threshold of 0.4. Modules most strongly related with HCC clinical traits were selected as key modules for further study. Finally, intersection analysis was performed among predicted ICT-target genes, HCC-associated disease genes, DEGs, and key module genes, and the overlapping genes, defined as ICT-related DEGs in HCC, were subjected to subsequent functional and prognostic analyses.

#### 2.1.3 Functional enrichment analysis

FEA of the overlapping genes was performed utilizing the R package “clusterProfiler,” incorporating both Kyoto encyclopedia of genes and genomes (KEGG) and gene ontology (GO) pathway analyses (25). A significance threshold of *p* < 0.05 was employed, and the enriched GO terms and KEGG pathways were imaged utilizing the “ggplot2” and “enrichplot” packages to ensure clear and intuitive presentation of the results.

#### 2.1.4 Protein–protein interaction network construction

To construct the PPI network, the overlapping genes were uploaded to the STRING database (https://www.string-db.org), restricting the Homo sapiens’ species and applying a confidence score threshold of 0.4. Following network optimization through the removal of isolated nodes, the interaction data were exported in TSV format and imported into Cytoscape 3.10.2 for visualization. Node color gradients were assigned based on degree values, reflecting the topological importance of each protein within the network.

#### 2.1.5 Cox regression and machine learning

To identify prognostic biomarkers associated with ICT in HCC, univariate Cox regression analysis was performed on genes shared by a cohort of TCGA HCC patients who had complete clinical information and survived more than 30 days. To enhance the accuracy of key ICT-related gene selection, four complementary machine learning approaches were employed, including SVM-RFE, XGBoost, GMM, and RF.

The XGBoost algorithm, implemented via the “xgboost” R package, was initially applied to iteratively construct decision trees, correcting previous errors to improve model performance and identify critical predictive genes, with features exhibiting gain values greater than zero considered important (26). Subsequently, GMM analysis was carried out utilizing the “SimDesign” package to determine the model with the highest classification accuracy and its associated genes; this approach models gene expression data as a combination of multiple Gaussian distributions, capturing complex underlying biological patterns (27). Following this, the SVM-RFE technique was employed through the “e1071” R package to iteratively remove less significant features, thereby refining the optimal gene set (28). Additionally, the RF algorithm, applied via the “randomForest” package, generated multiple decision trees whose aggregated predictions enhanced model accuracy and stability, with genes showing a mean decrease Gini (MDG) index greater than 2.0 designated as essential (29). Ultimately, genes consistently identified by all four machine learning methods were classified as key prognostic ICT-related genes in HCC and selected for subsequent downstream analyses.

#### 2.1.6 Development of a risk scoring model

To systematically identify ICT-associated prognostic biomarkers, we employed LASSO Cox regression analysis and Kaplan-Meier (K-M) survival analysis within the TCGA-LIHC cohort to evaluate the prognostic potential of key genes. Genes identified through both LASSO Cox regression and K-M survival analysis were defined as core prognostic genes, and their significance in HCC was further evaluated using the “glmnet” R package, focusing on OS as the primary outcome. The optimal penalty parameter (λ) was measured through ten-fold cross-validation and consistently applied across the analysis. Gene coefficients were extracted using the “coef” function to quantify each gene’s contribution to the model, and patient risk scores were calculated as risk score = Σ (Xi × Yi), where Xi represents the regression coefficient of gene i, Yi denotes its expression level. Patients were stratified into lower- and higher-risk groups based on the median risk score, and the model’s prognostic performance was gauged by comparing OS between these groups using K–M analysis. Furthermore, time-dependent receiver operating characteristic (ROC) curve analysis was directed with the “timeROC” package to calculate the predictive accuracy of the model for patient survival outcomes (30).

#### 2.1.7 Drug sensitivity analysis and immune infiltration analysis

The genomics of drug sensitivity in cancer database and the “oncoPredict” R package were employed to evaluate the relationship between sorafenib sensitivity and the expression levels of ICT-related prognostic genes, with the aim of predicting cellular responsiveness to pharmacological treatment (31).

The “CIBERSORT” algorithm was utilized to quantify the relative proportions of 22 distinct immune cell subtypes within the samples. Differences in immune cell composition across higher- and lower-risk groups were measured utilizing the Wilcoxon rank-sum test. Additionally, Spearman’s rank correlation analysis was conducted to evaluate the relationships, whether positive or negative, between the expression levels of prognostic genes and the proportions of specific immune cell populations.

#### 2.1.8 Validation of ICT-target binding through molecular docking

The SDF format file of ICT was first obtained from the PubChem database, and the protein data bank (PDB) format file of the corresponding core target receptor protein was retrieved from the PDB (http://www.rcsb.org/). Subsequently, the ligand structure was converted to the mol2 format using Chem3D 14.0, while the receptor proteins underwent preprocessing steps such as removal of were preprocessed in PyMOL 2.6.0 by removing water molecules and addition of adding hydrogen atoms in PyMOL 2.6.0. Molecular docking between ICT and the receptor proteins was then performed using AutoDockTools 1.5.7. The binding energy of each protein–ligand pair complex was recorded, calculated, and the resulting complex conformations were saved in pdbqt format. Finally, three-dimensional the 3D visualization and graphical representation analysis of the molecular docking results were conducted using carried out in PyMOL software to intuitively assess clearly illustrate the interaction modes.

#### 2.1.9 Validation of binding affinity using molecular dynamics simulations

MD simulations of the small molecule-protein complexes were performed using GROMACS 2020.6. The simulations were conducted under constant temperature (300 K) and pressure (1 bar) conditions (32). The topology of the small-molecule ligand was generated using the GAFF2 force field, while the protein was parameterized with the CHARMM36 force field (33). The protein receptor was solvated in a TIP3P water model, and sodium and chloride ions were introduced to neutralize the system’s net charge. Energy minimization was performed using the steepest descent method followed by the conjugate gradient approach. Subsequently, a 100,000-step equilibration was carried out under both isothermal–isochoric (NVT) and isothermal–isobaric (NPT) ensembles. Finally, two independent systems were subjected to 1,000,000-step MD simulations (corresponding to 100 ns) under periodic boundary conditions. The resulting trajectories were analyzed using the built-in tools of GROMACS.

### 2.2 Experimental verification

#### 2.2.1 Cell lines and materials

Human HCC HepG2 cells (CL-0103), Huh7 cells (CL-0120), fetal bovine serum (Cat. No. 164210-50), DMEM higher-glucose medium (Cat. No. PM150210), and 100× penicillin–streptomycin solution (Cat. No. PB180120) were purchased from Procell, while 0.25% trypsin–EDTA solution containing phenol red (Cat. No. SL6020) was obtained from Coolaber. ICT (purity ≥98%, Cat. No. A1458605) was procured from AmBeed. All reagents and cell lines were sourced from their respective companies in China or the USA.

#### 2.2.2 Cell culture and subculture

All experimental procedures followed standard cell culture protocols. HepG2 and Huh7 cells were maintained in DMEM added with 10% FBS and 1% streptomycin-penicillin solution and incubated in a humidified environment with 5% CO_2_ at 37°C. Cells were subcultured upon reaching approximately 70–80% confluence to ensure optimal growth conditions. During subculturing, the culture medium was eliminated, cells were rinsed with PBS, and subsequently detached using 0.25% trypsin–EDTA before being counted for downstream experiments.

#### 2.2.3 Cell viability assay

To assess HCC cell viability Following ICT treatment, HepG2 and Huh7 cells were seeded in the logarithmic growth phase (LGP) into 96-well plates at a density of 5 × 10³ cells per well. After cell adherence, the culture medium was substituted with fresh medium comprising various concentrations of ICT HepG2 cells were treated with 0, 3.25, 7.5, 15, 30, and 60μM ICT, while Huh7 cells were exposed to 0, 5, 10, 20, 40, and 80μM ICT for 24 and 48 hours. Post-treatment, the medium was removed, and a working solution was prepared by mixing complete culture medium with the cell counting kit-8 (CCK-8) reagent (Catalog No. BMU106-CN, Abbkine, China) at a 10:1 (v/v) ratio. This solution was carefully poured to each well, which was then followed by an additional 1-hour incubation. Absorbance was then measured at 450 nm utilizing a Varioskan Lux microplate reader (Thermo Scientific) to estimate cell viability.

#### 2.2.4 Wound healing assay

For the WHA, straight reference lines were drawn on the bottom of a 6-well plate using a marker pen. Cells in the LGP were seeded at a density of 6 × 10^4^ cells per well and incubated under standard culture conditions for 24 hours until reaching up to 80% confluence. A uniform scratch wound was then created perpendicular to the marked lines using a sterile 1 mL pipette tip. Following wounding, culture medium containing different concentrations of ICT (0, 7.5, 15, and 30 μM or 0, 5, 10, and 20 μM) was added to the respective wells. Images of the wound area were captured at 0-, 24-, and 48-hours post-scratch, and the wound closure was quantitatively analyzed utilizing ImageJ software.

#### 2.2.5 Immunofluorescence staining experiment

Double-target immunofluorescence staining was performed using the tyramide signal amplification (TSA) technique. Briefly, cells were permeabilized for 10 minutes at room temperature with 0.1% Triton X-100 (T8200, Solarbio, China) and rinsed three times with PBS. Nonspecific binding was blocked by incubating the cells with 10% goat serum (PN0038, pinuofei Biotech, China) at 37°C for 30 minutes. The cells were then incubated with primary antibodies (Wuhan Sanying, China: Fatty acid-binding protein 5 (FABP5)/12348-1-AP/1:200, UCK2/66822-1-IG/1:200, carbonic anhydrase 9 (CA9)/11071-1-AP/1:200, CYP2C9/16546-1-AP/1:500), the same antibodies used for Western blotting (WB), either for 2 hours at 37 °C or for the entire night at 4°C. Following primary incubation, HRP-conjugated goat anti-rabbit IgG secondary antibody (PN0046, pinuofei Biotech) was applied at 37°C for 1 hour. After extensive washing with PBST (it is PBS, which has 0.1% Tween-20), TSA was performed sequentially with TYR-488 (PN0100, pinuofei Biotech) and TYR-555 (PN0101, pinuofei Biotech) at 37°C for 30 minutes each. Nuclei were counterstained with DAPI (PN0015, pinuofei Biotech) for 5 minutes, and samples were mounted using an anti-fluorescence quenching mounting medium (PN0024, pinuofei Biotech) and stored at 4°C in the dark. Fluorescence images were acquired using a fluorescence microscope, and the fluorescence intensity was quantitatively analyzed using ImageJ software. Finally, the relative protein expression level was determined by comparing the mean fluorescence intensity of the target protein to that of DAPI. PBST was used for all washing steps throughout the procedure, and working concentrations of all antibodies and reagents were pre-optimized in preliminary experiments to ensure optimal staining efficiency and signal specificity.

#### 2.2.6 qRT-PCR analysis

Total RNA was extracted from cells utilizing TriQuick Total RNA Extraction Reagent (R1100, Solarbio, China) and purified via a precipitation method employing chloroform (CAS: 67-66-3, Sinopharm, China) and isopropanol (CAS: 80109218, Sinopharm), followed by washing with anhydrous ethanol (10009218, Sinopharm). The purified RNA was dissolved in DEPC-treated water (R0022, Beyotime, China) for storage. Genomic DNA was eliminated, and complementary DNA was synthesized utilizing the Evo M-MLV RT Mix Kit with gDNA Clean for quantitative PCR (qPCR) Ver.2 (AG11728, Accurate Biology, China). qPCR was performed using PerfectStart^®^ Green qPCR SuperMix (+Dye II, AQ602, TransGen, China) under the following thermal cycling conditions: initial denaturation at 94°C for 2 minutes, 45 cycles of amplification (94°C for 5 seconds, 60°C for 30 seconds), and a final melting curve analysis from 65°C to 95°C with 0.5°C increments every 5 seconds. Relative expression levels of the target genes were calculated utilizing the 2^−ΔΔCt^ method, with *β*-actin as the internal reference. All experiments included three biological replicates and three technical replicates. Primer sequences are provided in **Table 1**.

**Table 1:** Primers sequences used in this study.

Targets	Sequences	
	Forward:5’-3’	Reverse:5’-3’
UCK2	CAGCTAGCGGCAAGAGGTAA	TGCTCAGTCCCAAAGCTGAG
CA9	CCAGGGTGTCATCTGGACTG	TCAGCTGTAGCCGAGAGTCA
FABP5	TCTTGTACCCTGGGAGAGAAGT	CCACTCCTGATGCTGAACCA
CYP2C9	GGGGCATTATCCATCTTTCACT	ACTCTCCGTAATGGAGGTCG

#### 2.2.7 Western blotting

Cells were lysed utilizing RIPA buffer for 30 minutes. The lysates were centrifuged for 15 minutes at 12,000 rpm to collect the supernatant, and protein concentrations were quantified using a BCA protein assay kit (E-BC-K318-M, Elabscience, China). Sample concentrations were then adjusted to ensure uniform protein loading based on the standard curve. Twenty micrograms of protein per sample were separated on a 4–20% Bis-Tris precast gel at 160 V for 40 minutes and transferred onto a PVDF membrane at a constant current of 200 mA. Membranes were blocked with 5% skim milk in TBST at room temperature for 1 hour and incubated overnight at 4°C with primary antibodies diluted 1:1000, alongside GAPDH as the internal reference (1:5000, YM3029, Immunoway, China). Following three washes with TBST, HRP-conjugated goat anti-rabbit IgG (RS0002, Immunoway; 1:5000) was applied for 1 hour at room temperature. After additional washes, protein bands were imaged utilizing ECL chemiluminescent reagent, and images were captured with an automatic chemiluminescence imaging system.

#### 2.2.8 Statistical analysis

All data are expressed as mean ± standard deviation (SD). Moreover, statistical analyses were carried out utilizing GraphPad Prism 8.0, with one-way or two-way ANOVA applied for multiple group comparisons. Statistical significance was denoted as follows: *p < 0.05, **p < 0.01, ***p < 0.001, and ****p < 0.0001.

## 3 Result

### 3.1 Investigation utilizing network pharmacology and bioinformatics approaches

#### 3.1.1 Discovery of ICT-related DEGs in HCC

**Figure 3:**
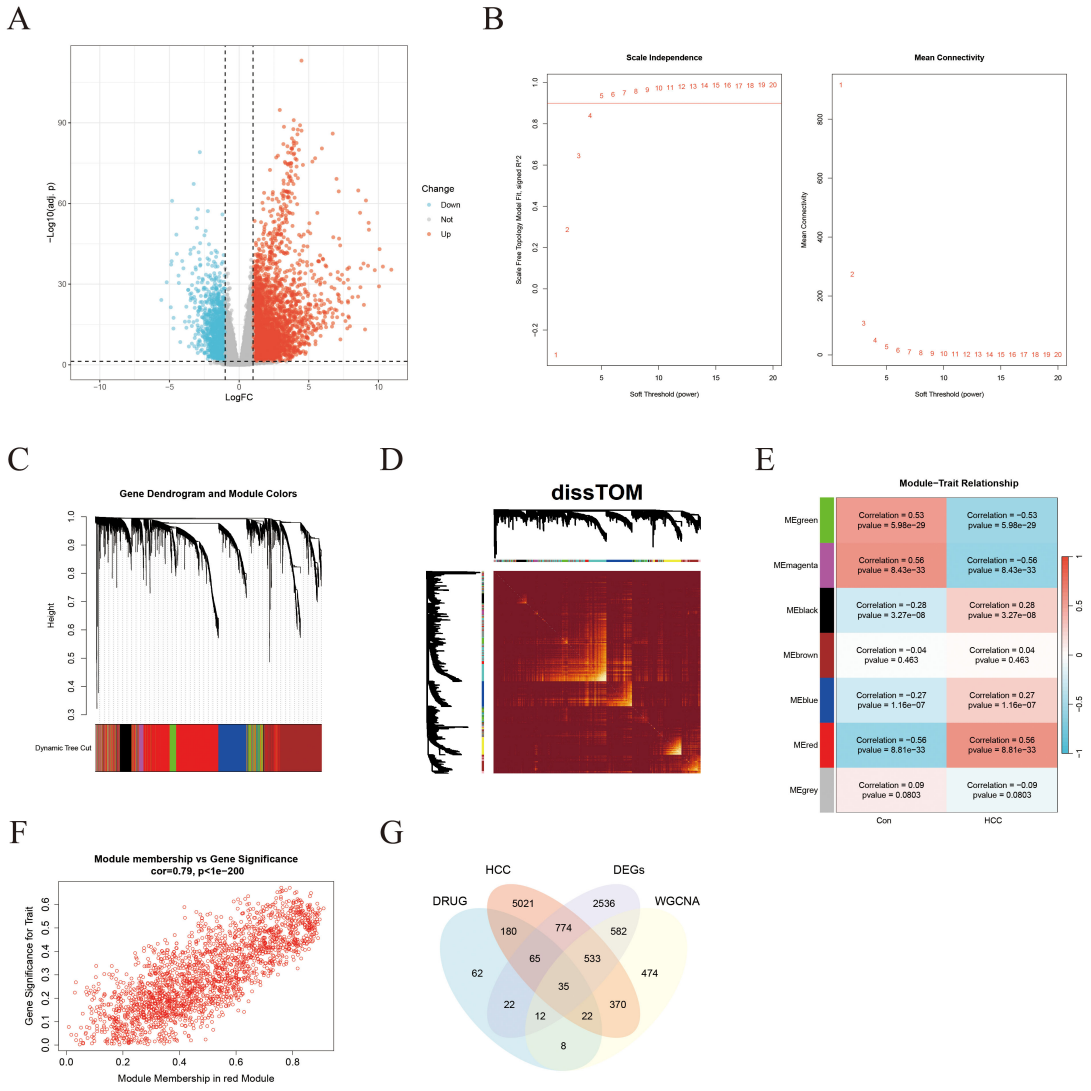
Identification of ICT-associated DEGsin HCC through integrated DEA and WGCNA. **(A)** Volcano plot illustrating DEGs between HCC tumor tissues and adjacent normal tissues in the TCGA-LIHC cohort. **(B)** Network topology plots depicting scale-free fit and mean connectivity across varying soft-thresholding powers. **(C)** Hierarchical clustering dendrogram of DEGs. **(D)** Heatmap showing the dissTOM among all identified gene modules. **(E)** Correlation heatmap between gene modules (represented by colors) and clinical traits. **(F)** Scatter plot demonstrating the positive correlation between gene significance (GS) and module membership (MM) for the red module in tumor tissues. **(G)** Venn diagram illustrating the intersection among HCC DEGs, ICT-related targets, HCC-derived module genes, and HCC-associated disease genes.

After excluding outliers from the TCGA-LIHC dataset, a total of 19,938 protein-coding genes were obtained. DEA revealed that 3,335 genes were significantly upregulated in HCC tissues relative t controls, while 1,204 genes were downregulated (**Figure 3A**). WGCNA was then applied to establish co-expression networks and detect gene modules in both control and HCC groups. The top 25% of genes displaying the maximum variance, totaling 4,985 genes, were selected for WGCNA. A soft-thresholding power (*β*) of 5 (*R²* = 0.9) was chosen to achieve a scale-free network topology, leading to the identification of seven distinct co-expression modules, each represented by a different color (**Figures 3B, C**). The dissTOM matrix heatmap, visualized using the dynamic tree cut algorithm, further illustrated the modular structure (**Figure 3D**). Clinical features (control versus HCC status) were incorporated to evaluate module–trait associations, revealing that the red module, comprising 2,036 genes, displayed the strongest and most statistically significant correlation with HCC (**Figure 3E**). A scatter plot confirmed this association, demonstrating a strong positive correlation between module eigengene values and HCC status (**Figure 3F**). Finally, integration of potential ICT target genes, HCC-related disease genes, DEGs, and key module genes via a Venn diagram identified 35 overlapping genes (see **Supplementary Table 1**), which were designated as ICT-linked DEGs in HCC (**Figure 3G**).

#### 3.1.2 Enrichment analysis and PPI network construction of intersection genes

**Figure 4:**
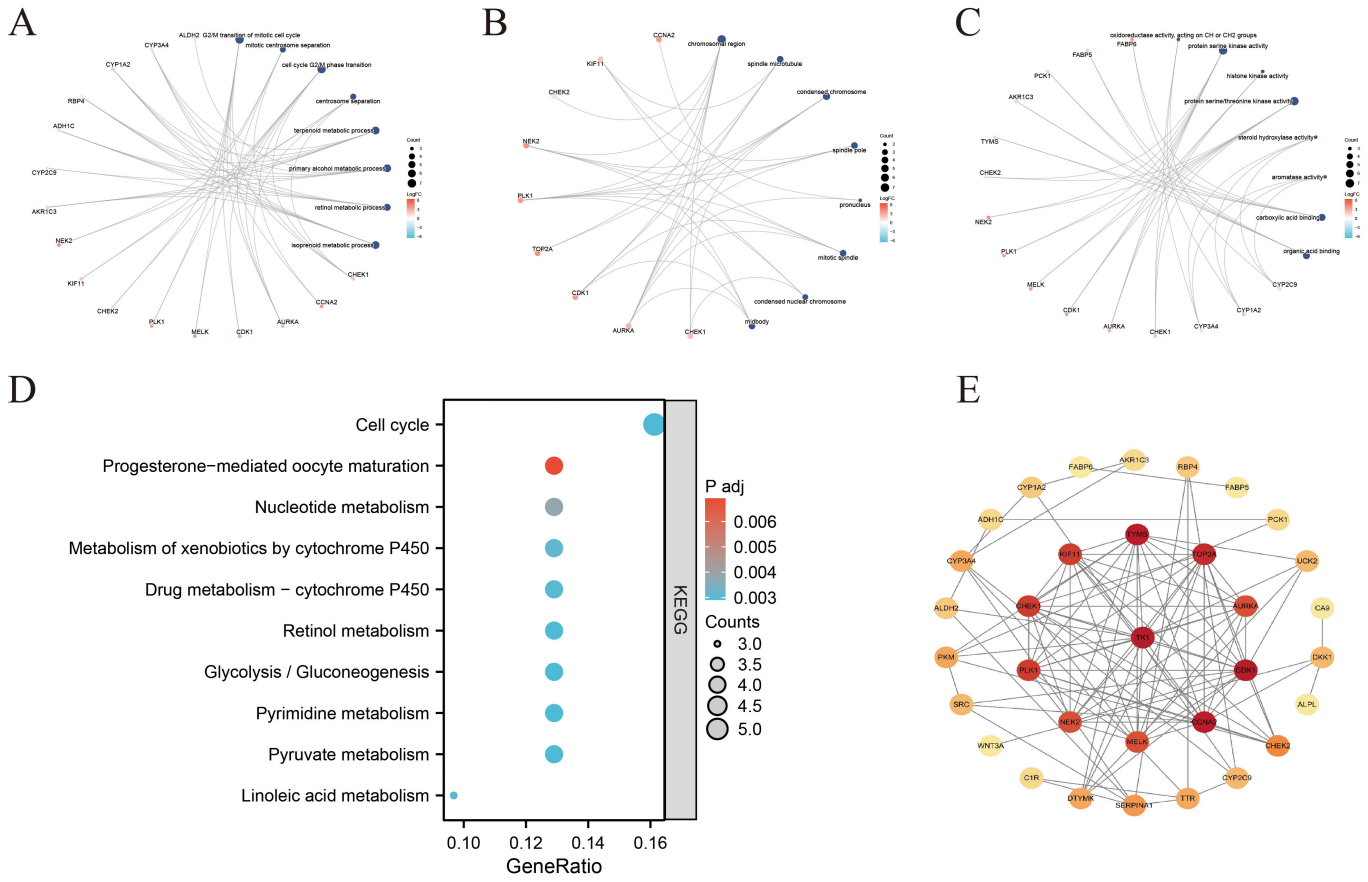
Functional enrichment analysis and PPI network construction of ICT-associated differentially expressed genes in HCC. **(A–C)** Network diagrams illustrating the top 8 enriched categories in BPs, CCs, and MFs. **(D)** Bubble plot showing the top ten suggestively enriched KEGG pathways. **(E)** PPI network of ICT-associated DEGs in HCC.

Functional enrichment analyses were carried out on the 35 overlapping genes, encompassing GO and KEGG pathway annotations. In the BP category, the genes were predominantly enriched in pathways associated with the G2/M phase transition of the cell cycle, terpene metabolism, primary alcohol metabolism, and retinol metabolism (**Figure 4A**). For cellular components (CC), the genes were mainly linked to structures such as chromatin, spindle microtubules, and condensed chromatin (**Figure 4B**). Regarding molecular functions (MFs), momentous enrichment was detected in oxidoreductase activity, carboxylic acid binding, organic acid binding, and protein serine/threonine kinase activity (**Figure 4C**). KEGG pathway analysis indicated that ICT may exert therapeutic effects on HCC by modulating key signaling pathways, including the cell cycle, nucleotide metabolism, glycolysis/gluconeogenesis, and drug metabolism pathways (**Figure 4D**). To further investigate interactions among these target proteins, the genes were analyzed utilizing the STRING database to exclude non-interacting targets, and the resulting dataset was imported into Cytoscape 3.10.2 to build a PPI network (**Figure 4E**).

#### 3.1.3 Identification of core genes associated with ICT and construction of a prognostic model

**Figure 5:**
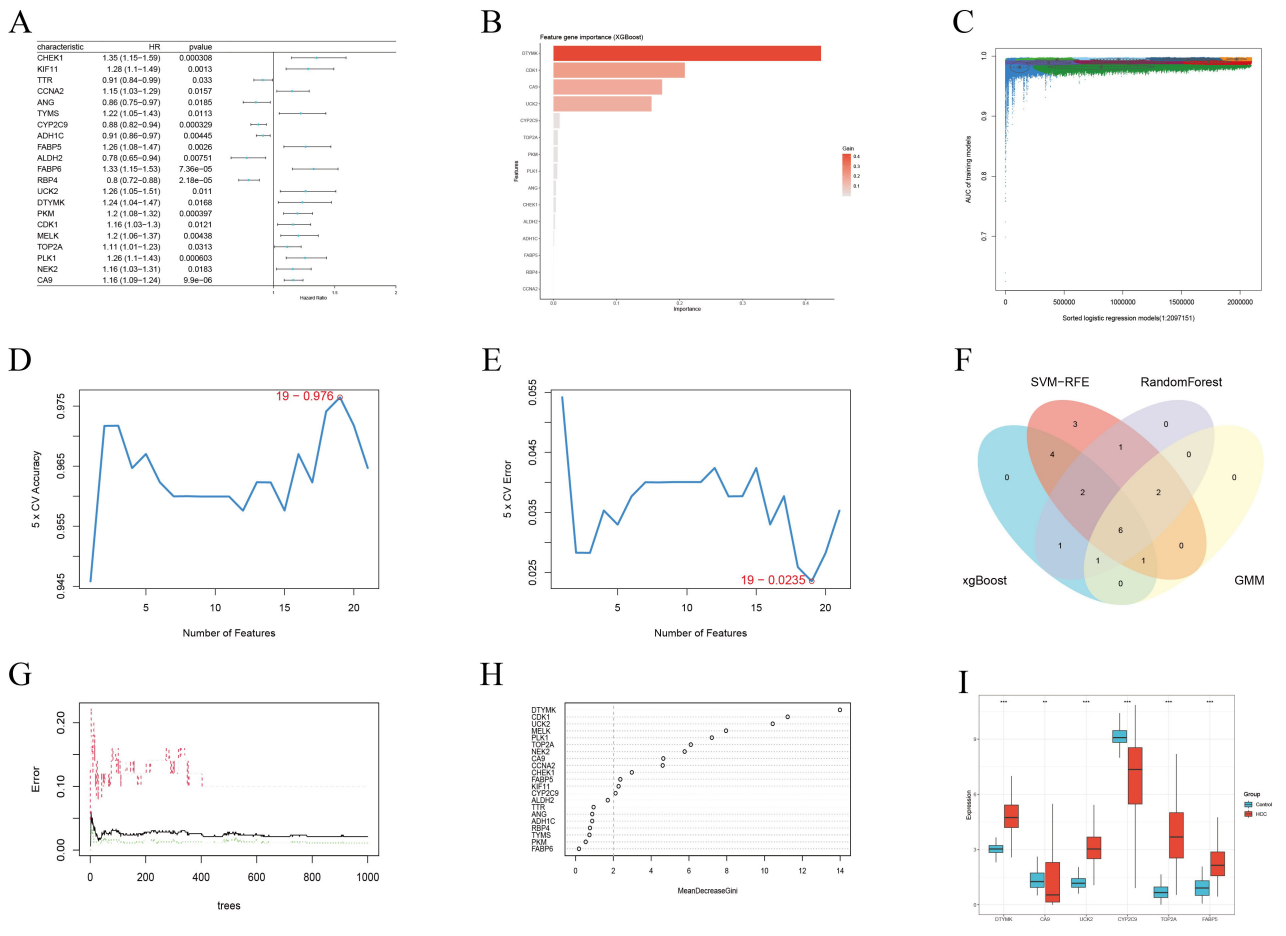
Candidate prognostic genes were identified through integration of Cox regression analysis and machine learning algorithms. **(A)** Univariate Cox regression analysis identified 21 genes significantly associated with OS. **(B)** Core diagnostic markers selected using the XGBoost algorithm (n=15). **(C)** Identification of significant genes via GMM regression analysis (n=10). **(D, E)** Feature genes detected by the SVF-RFE method (n=19). **(F)** Venn diagram showing the intersection of results from four MLAs used to define candidate prognostic genes. **(G, H)** Key predictive features selected by the random forest algorithm (n=13). **(I)** Box plots depicting the expression levels of six candidate prognostic genes in the TCGA-LIHC cohort. * < 0.05, ** < 0.01, *** < 0.001.

Univariate Cox regression analysis of the 35 overlapping genes identified 21 ICT-associated genes with statistically significant prognostic value (**Figure 5A**). To further refine key prognostic candidates, multiple machine learning algorithms (MLAs) were applied. Using XGBoost, 15 core genes with gain values greater than zero were identified (**Figure 5B**). GMM regression analysis, performed over 221 iterations, selected a model with optimal predictive performance (area under the curve (AUC)≈ 0.998) comprising 10 core genes (**Figure 5C**). The SVM-RFE approach identified 19 feature genes (**Figures 5D, E**), while the RF algorithm, integrating multiple decision trees, highlighted 13 genes with MDG values greater than 2.0 as key prognostic features (**Figures 5G, H**). Intersection analysis of these gene sets yielded six overlapping genes as key candidate prognostic markers (**Figure 5F**), whose expression levels were compared between control and HCC tissues (**Figure 5I**).

**Figure 6:**
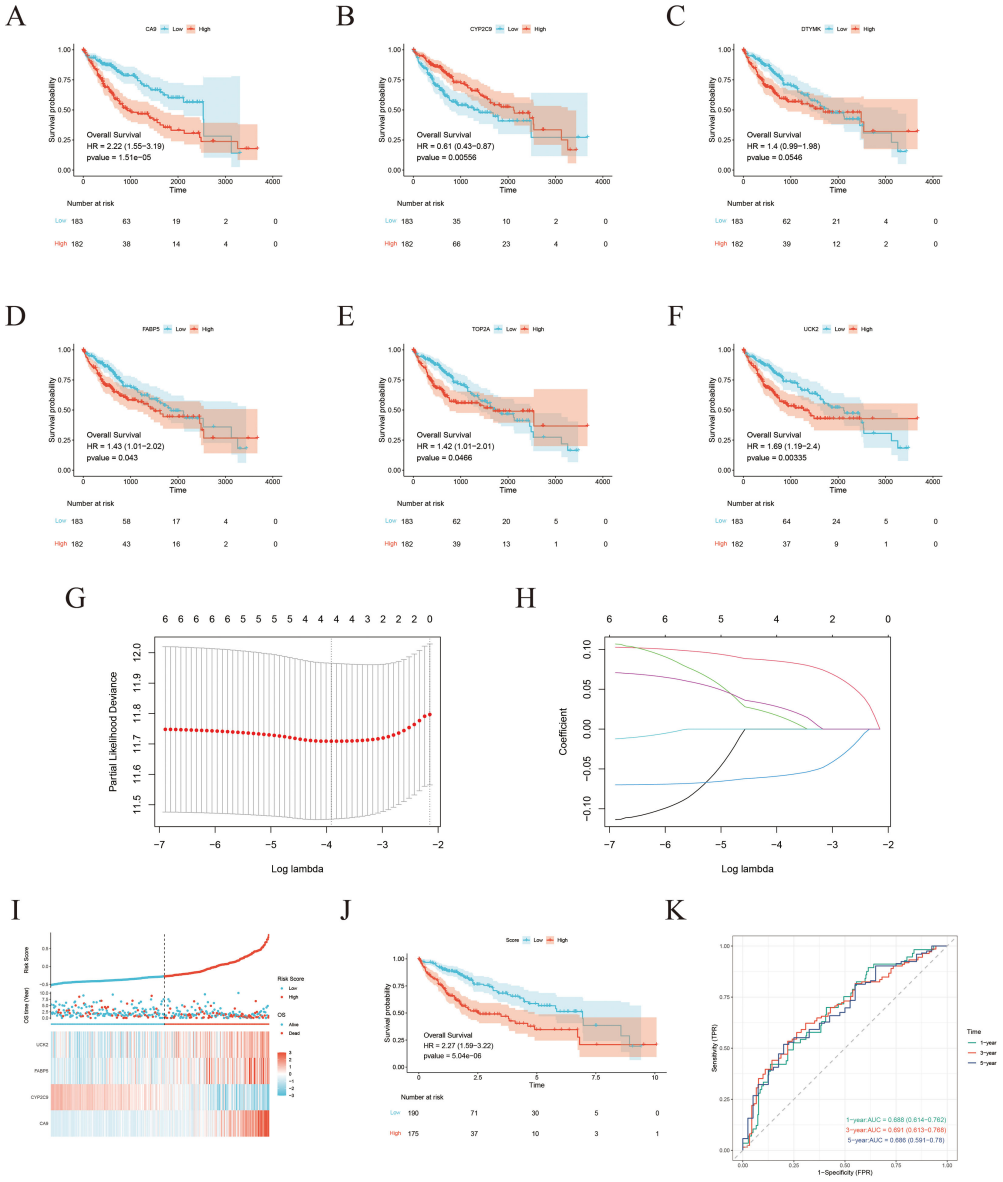
Development of a risk score model for prognostic prediction. **(A–F)** K-M survival curves demonstrating the prognostic value of CA9, CYP2C9, DTYMK, FABP5, TOP2A, and UCK2 expression levels in the TCGA-LIHA cohort. **(G)** Establishment of the prognostic model using LASSO Cox regression analysis. **(H)** LASSO coefficient profile across varying lambda values. **(I)** Risk score distribution, survival status, and expression heatmap ofthe four ICT-associated genes in the TCGA-LIHA cohort. **(J)** Kaplan–Meier survival analysis comparing OS between higher-risk and lower-risk groups. **(K)** Time-dependent ROC curves evaluating the predictive performance of the risk score model for 1-year, 3-year, and 5-year survival in theTCGA-LIHC cohort.

To construct a strong prognostic model, the six candidate genes were further analyzed utilizing K–M survival analysis and LASSO Cox regression. K-M analysis revealed that five genes (UCK2, FABP5, CA9, CYP2C9, and TOP2A) met the significance criterion (*p* < 0.05), indicating their expression levels were significantly associated with OS (**Figures 6A–F**). Ten-fold cross-validation was applied to minimize overfitting, and LASSO regression identified four genes, UCK2, FABP5, CA9, and CYP2C9, as significant predictors (**Figures 6G, H**). Subsequently, based on the intersection of results from these two analytical approaches, a four-gene risk score model was established via LASSO analysis (**Figure 6I**). The risk score measured as follows: Risk Score = 0.0849 × CA9 + 0.0151 × UCK2 – 0.0585 × CYP2C9 + 0.0267 × FABP5 expression levels (**Figure 6I**).

Using the four-gene risk score, HCC patients with available clinical data from the TCGA database were stratified into lower-risk and higher-risk groups. K–M survival analysis revealed that patients in the lower-risk group had markedly improved OS compared with those in the higher-risk group (**Figure 6J**). The predictive performance of the model was further assessed using time-dependent ROC curve analysis, yielding AUC values of 0.688, 0.691, and 0.686 at 1, 3, and 5 years, respectively, demonstrating the model’s moderate prognostic accuracy (**Figure 6K**).

#### 3.1.4 Analysis of drug sensitivity and immune cell infiltration

**Figure 7:**
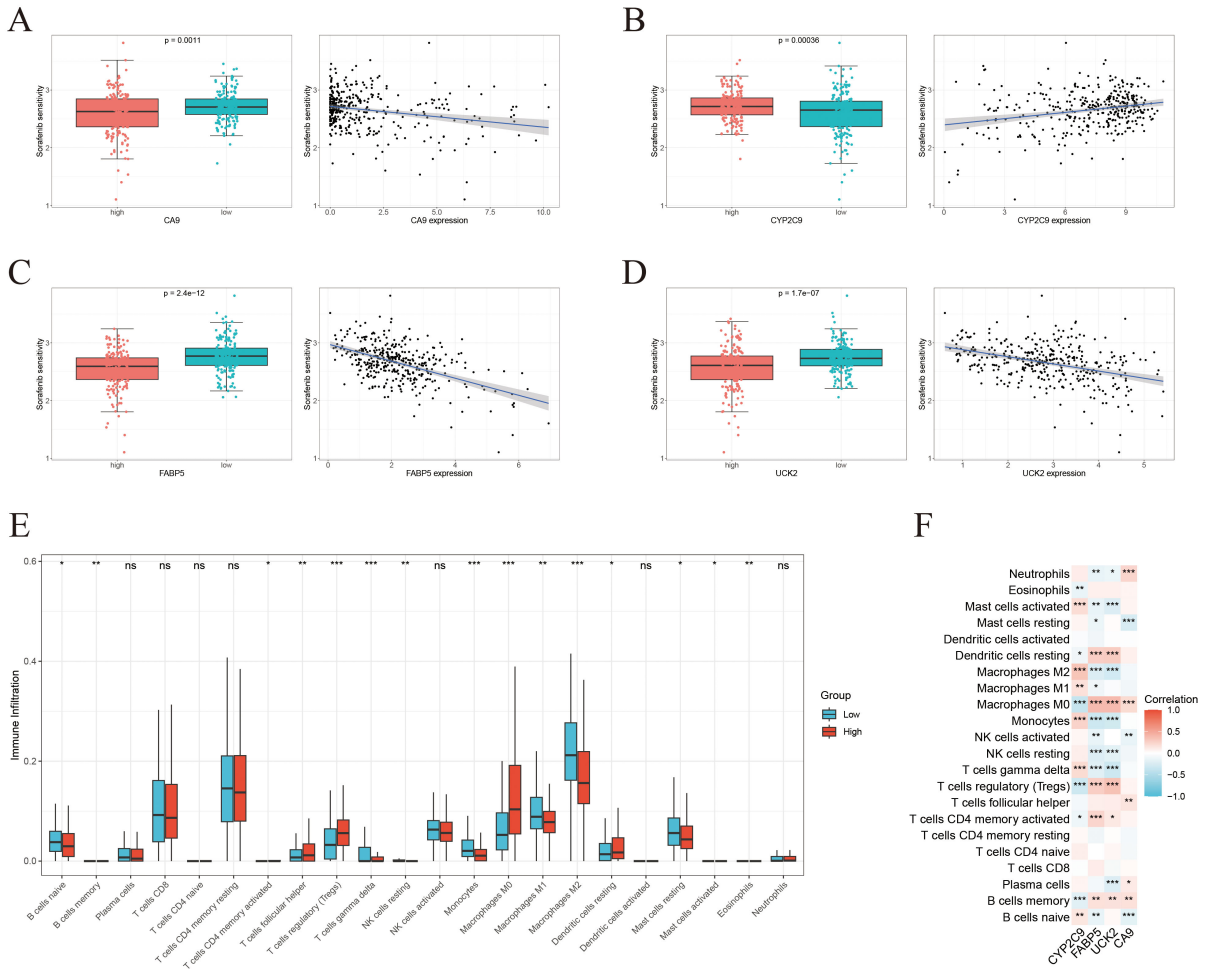
Drug sensitivity profiling and immune infiltration characterization. **(A-D)** Individual analysis of the correlation between the expression level of each core gene (CA9, CYP2C9, FABP5, UCK2) and sorafenib sensitivity. **(E)** Comparison of immune cell infiltration proportions between the high-risk and low-risk groups defined by the four-gene risk score. **(F)** Correlation analysis between the continuous risk score and the infiltration levels of various immune cells. * < 0.05, ** < 0.01, *** < 0.001.

The link between gene expression levels and sorafenib sensitivity was assessed using the “oncoPredict” package. Box and scatter plots demonstrated significant correlations between the expression of four key genes and drug sensitivity, with all *p*-values < 0.01, signifying their potential as predictive biomarkers for sorafenib response. Notably, CYP2C9 expression exhibited a positive correlation with sorafenib sensitivity, whereas UCK2, FABP5, and CA9 showed negative correlations (**Figures 7A–D**).

To investigate immunological implications, immune infiltration was analyzed using the CIBERSORT algorithm, revealing significant variances in the proportions of 15 distinct immune cell populations between higher-risk and lower-risk groups stratified by the four-gene risk score (**Figure 7E**). Further correlation analysis between ICT-related prognostic hub genes and immune cell infiltration indicated strong associations, particularly involving M0 macrophages, memory B cells, monocytes, and regulatory T cells (**Figure 7F**).

#### 3.1.5 Molecular docking results

**Figure 8:**
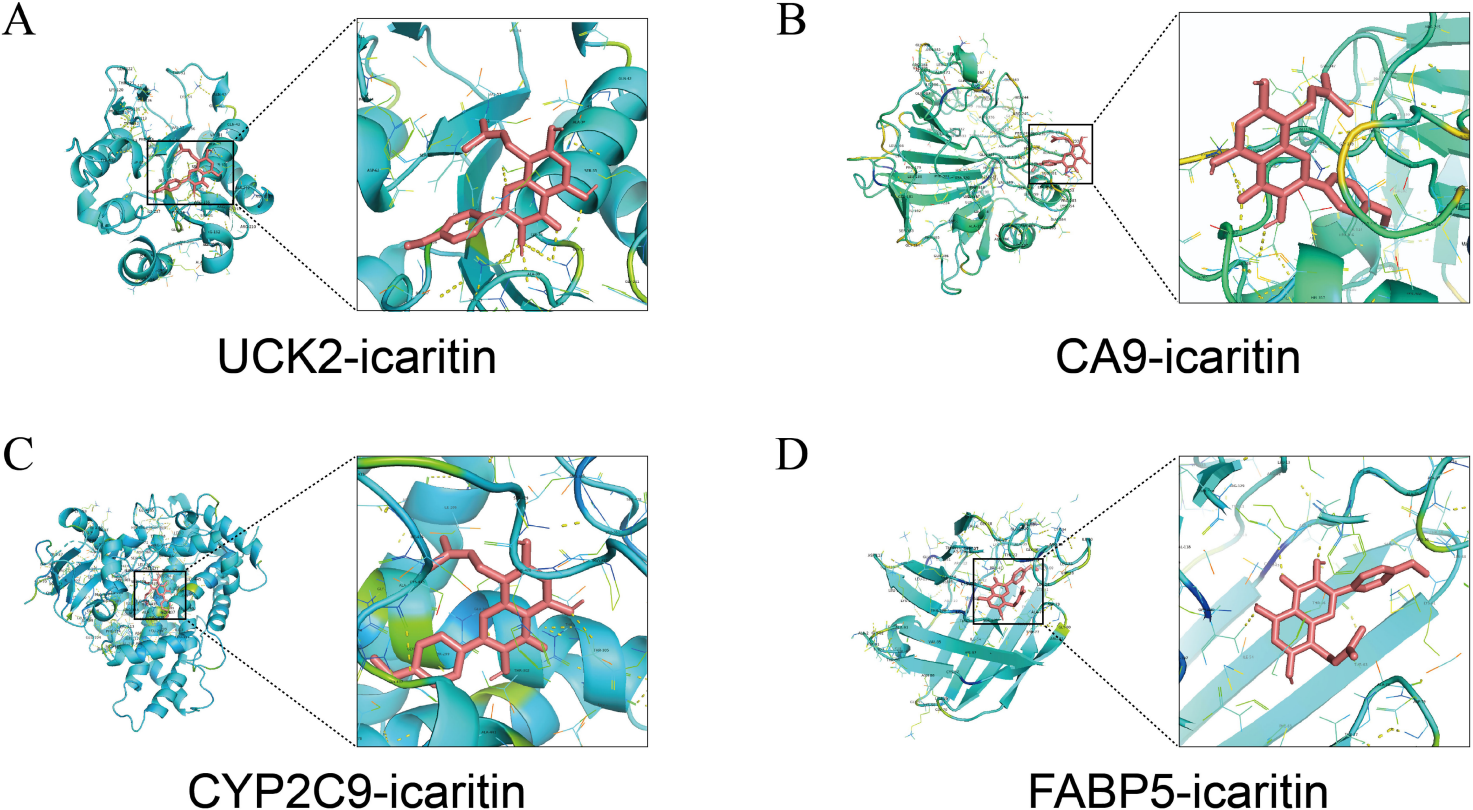
Molecular docking patterns between ICT and its target proteins. **(A)** UCK2. **(B)** CA9. **(C)** CYP2C9. **(D)** FABP5.

Molecular docking analysis revealed that ICT exhibited negative binding energy values with the key target proteins CA9, UCK2, FABP5, and CYP2C9 (**Table 2**), indicating direct interactions with amino acid residues within these proteins. Lower binding energies correspond to more stable and favorable molecular interactions, with values below −5.0 kJ/mol reflecting favorable binding affinity and values below −7 kJ/mol indicating strong binding activity. Notably, all four core targets demonstrated binding energies below −7 kJ/mol, suggesting their critical involvement in ICT’s therapeutic mechanism against HCC. The docking results were visualized in **Figure 8**. Molecular docking analysis revealed that ICT exhibited binding energies of -7.3, -7.5, -8.9, and -9.5 kJ/mol with the four core target proteins UCK2, CA9, CYP2C9, and FABP5, respectively. Among these, ICT showed the strongest binding affinity toward FABP5. Therefore, FABP5—the protein with the highest binding affinity—was selected for subsequent MD simulations to further validate and characterize the stability of the binding interaction.

**Table 2:** Molecular docking results of icaritin with core targets.

Compound	Target	PDB: ID	Binding Energy (KJ/mol)
icaritin	UCK2	1UJ2	-7.3
icaritin	CA9	6RQU	-7.5
icaritin	CYP2C9	5A5I	-8.9
icaritin	FABP5	7G0E	-9.5

#### 3.1.6 Validation of binding capacity through molecular dynamics simulations

Although a semi-flexible docking strategy was applied during the molecular docking process, this approach remains limited in accurately capturing the influence of real-world environmental factors on proteins, such as temperature, pressure, conformational flexibility, and solvent effects. To overcome these limitations, a 100-ns all-atom MD simulation of the FABP5–ICT complex was performed using GROMACS to comprehensively analyze the protein’s dynamic behavior and conformational stability upon ligand binding. Following the simulation, multiple structural parameters were systematically assessed using the software’s built-in trajectory analysis tools, including root mean square deviation (RMSD), root mean square fluctuation (RMSF), radius of gyration (Rg), hydrogen bond interactions, and the free energy landscape (FEL).

**Figure 9:**
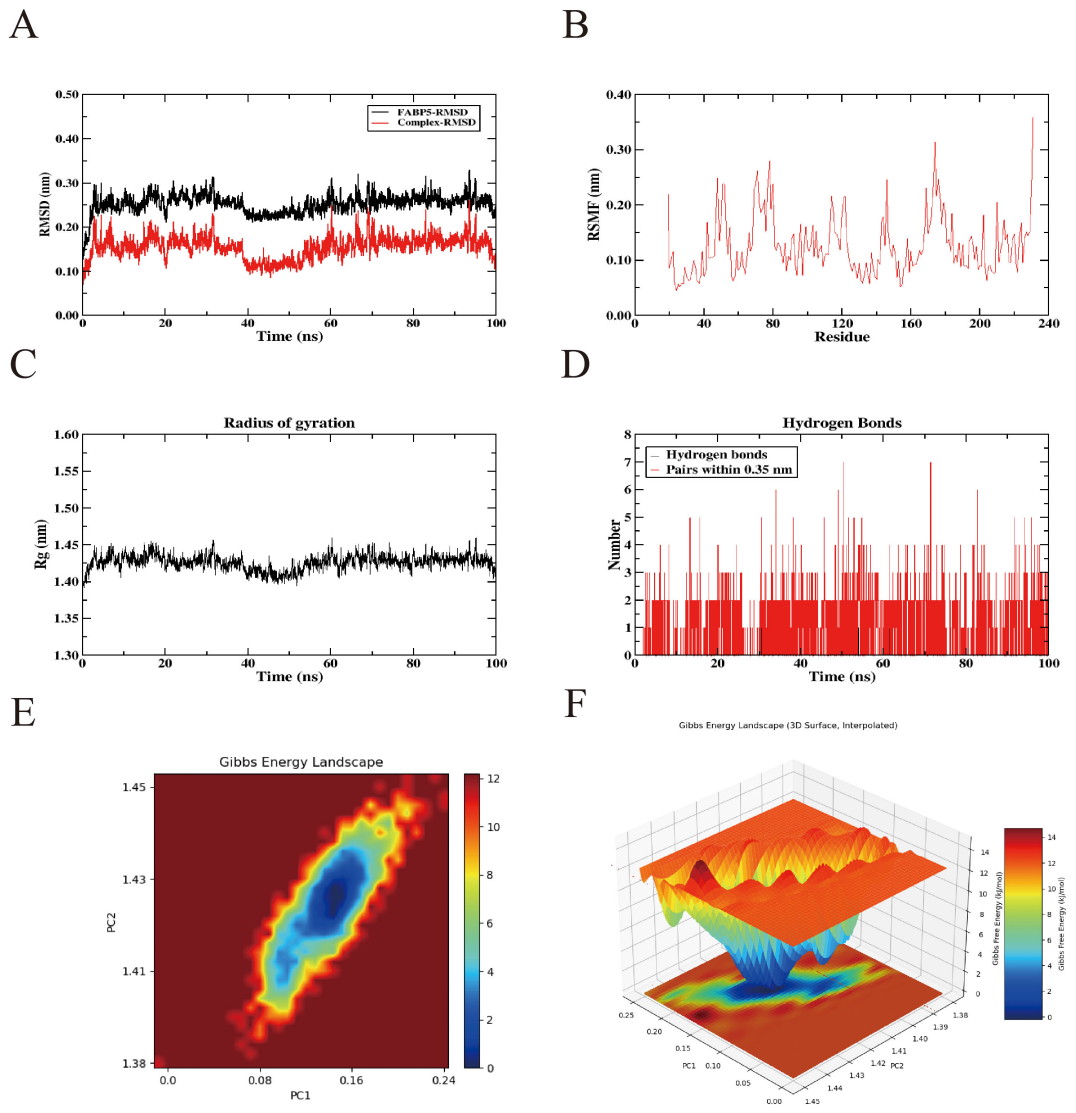
Molecular dynamics simulation results of the interaction between icaritin and the FABP5 protein. **(A)** Root-mean square deviation values of the FABP5 protein and the FABP5 protein–icaritin saponin complex. **(B)** Changes in protein flexibility during molecular dynamics simulation of icaritin. **(C)** Radius of gyration profile of the protein-icaritin complex. **(D)** Hydrogen bond dynamics during molecular dynamics simulations. **(E, F)** Two-dimensional and three-dimensional visualization of the free energy landscape.

The MD simulation results showed that the RMSD values of the protein–ligand complex fluctuated by less than 0.2 nm throughout the 100-ns trajectory, indicating that the complex maintained a relatively stable conformation under simulated physiological conditions (**Figure 9A**). RMSF analysis further revealed regions of higher residue flexibility, highlighting local conformational variations within the protein structure (**Figure 9B**). In parallel, the radius of gyration (Rg) exhibited only minor fluctuations, suggesting a compact overall structure and strong intramolecular cohesion (**Figure 9C**). Moreover, analysis of backbone hydrogen bonds indicated the formation of persistent and stable hydrogen bond interactions between FABP5 and ICT throughout the simulation period (**Figure 9D**). To further evaluate the conformational stability of the FABP5–ICT complex, principal component analysis was performed to extract the first two principal components from the RMSD and Rg data. These were integrated with the binding free energy, calculated using the Gibbs free energy equation, to construct the corresponding 2D and 3D FEL plots. The analysis revealed a distinct low-energy basin that persisted throughout the simulation, suggesting that the complex preferentially resides in energetically favorable and structurally stable conformations (**Figures 9E, F**). Collectively, these findings confirm the robust structural stability of the FABP5–ICT complex during dynamic simulation.

### 3.2 Experimental verification

#### 3.2.1 The influence of ICT treatment concentration and time on the survival rate of HCC cells

**Figure 10:**
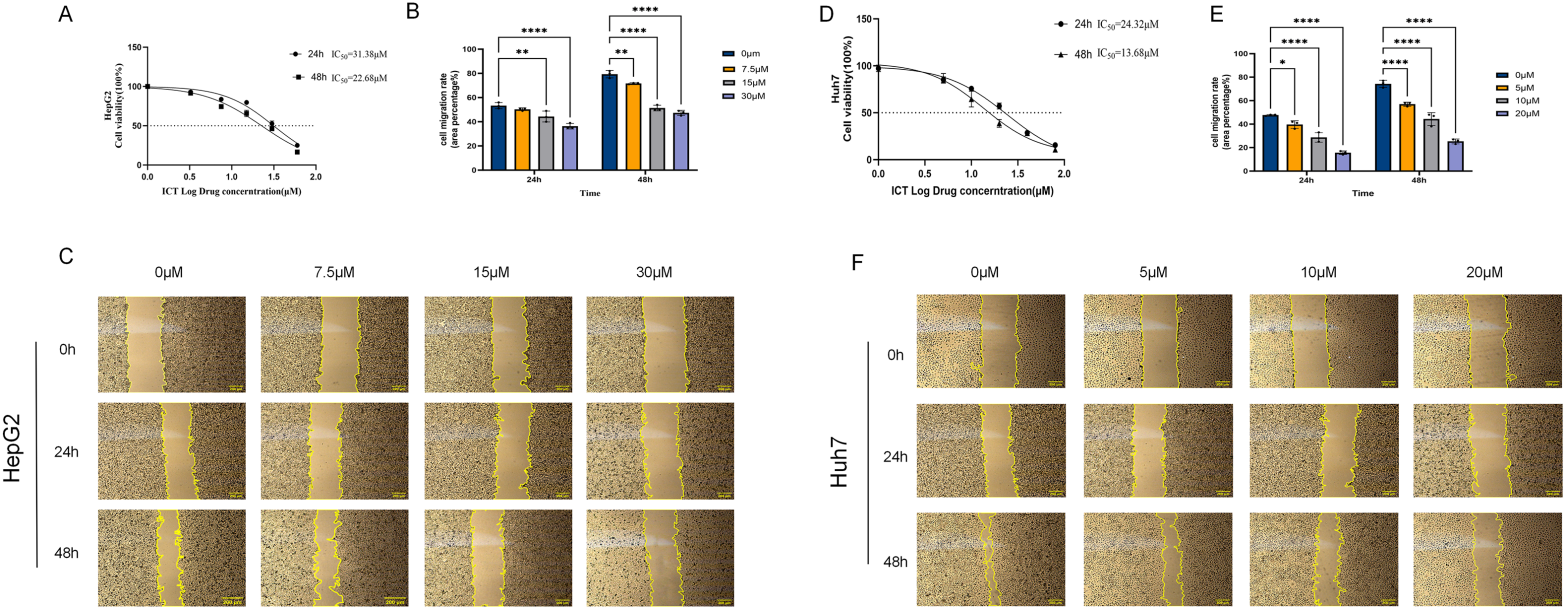
Effect of ICT on the proliferation and migration of hepatocellular carcinoma HepG2 and Huh7 cells. **(A)** Cytotoxic effect of ICT on HepG2 cells. **(B, C)** Quantification and representative images of the wound healing assay in HepG2 cells treated with various concentrations of ICT. **(D)** Cytotoxic effect of ICT on Huh7 cells. **(E, F)** Quantification and representative images of the wound healing assay in Huh7 cells treated with various concentrations of ICT. (*p < 0.05, **p < 0.01, ****p < 0.0001, scale bar: 200μm; data from three biological replicates, with three regions analyzed per replicate).

The results revealed that ICT inhibited HepG2 and Huh7 cells growth in a pronounced dose-dependent manner (DDM) and time-dependent manner (TDM) (**Figures 10A, D**). Specifically, cell viability decreased progressively with increasing ICT concentrations following 24-hour and 48-hour treatments. The IC50 values for HepG2 and Huh7 cells were 31.38 μM and 24.32 μM at 24 hours, respectively, and 22.62 μM and 13.68 μM at 48 hours, respectively, indicating enhanced cytotoxic activity with prolonged exposure. Based on the 24-hour IC50 values, three representative concentrations were selected for each cell line for subsequent experiments: 7.5 μM (approximately one-quarter of the IC50), 15 μM (approximately one-half of the IC50), and 30 μM (approximately the IC50) for HepG2 cells; and 5 μM (approximately one-quarter of the IC50), 10 μM (approximately one-half of the IC50), and 20 μM (approximately the IC50) for Huh7 cells.

#### 3.2.2 The influence of ICT on the migration ability of HCC cells

**Figure 11:**
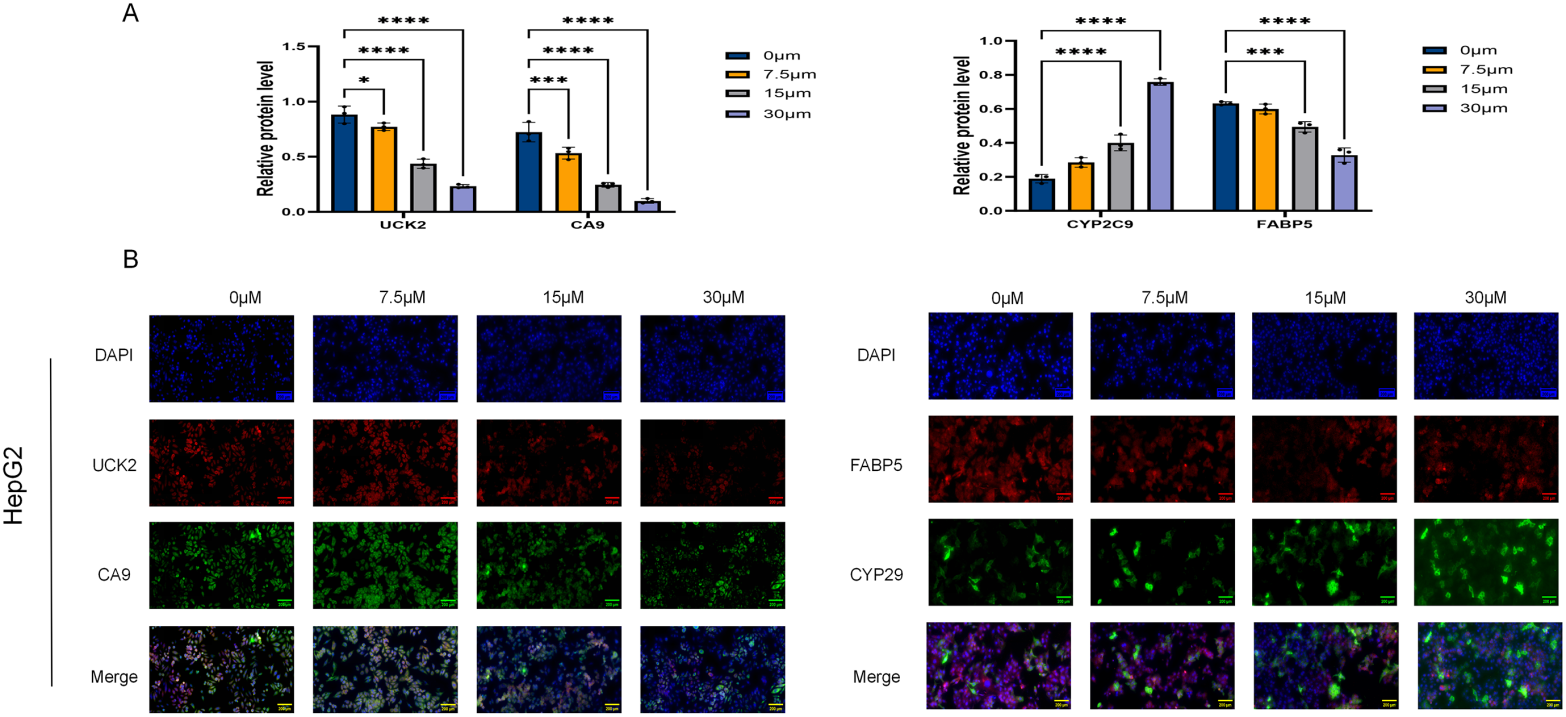
Quantitative immunofluorescence analysis of target protein expression in ICT-treated HepG2 cells. **(A)** Results of the quantitative immunofluorescence analysis. **(B)** Representative immunofluorescence images. (*p < 0.05, ***p < 0.001, ****p < 0.0001, scale bar:1:0.005; Data are presented as the mean ± standard deviation (Mean ± SD) from at least three independent experiments (n = 3.).

Experimental results demonstrated that ICT effectively inhibits HCC cell migration in a both DDM and TDM (**Figures 11B, C, E, F**). In HepG2 cells, after 24 hours of treatment with 0, 7.5, 15, and 30 μM ICT, the migration rates were 53%, 50%, 44% (*p* < 0.01), and 36% (*p* < 0.0001), respectively. This suppression was further enhanced after 48 hours, with the control group’s migration rate increasing to 79%, while the ICT-treated groups showed significantly reduced rates of 72% (*p* < 0.01), 52% (*p* < 0.0001), and 47% (*p* < 0.0001). Similarly, in Huh7 cells, 24-hour treatment with 0, 5, 10, and 20 μM ICT resulted in migration rates of 48%, 40% (*p* < 0.05), 28% (*p* < 0.0001), and 16% (*p* < 0.0001), respectively. After 48 hours, the control migration rate increased to 74%, whereas ICT treatment led to markedly suppressed migration rates of 57%, 44%, and 25% (all *p* < 0.01). Together, these findings indicate that ICT effectively inhibits hepatocellular carcinoma cell migration in a sustained and cumulative manner.

#### 3.2.3 The influence of ICT treatment on the expression of target protein-positive cells

These four genes were selected for *in vitro* validation based on a stepwise computational analysis. We first identified 35 ICT-HCC overlapping genes through network pharmacology and bioinformatics. Univariate Cox regression and four machine learning algorithms narrowed them to six candidate genes. Integrating Kaplan–Meier and LASSO Cox regression analyses, we defined a four-gene signature—UCK2, FABP5, CA9, and CYP2C9—that stratifies HCC patients into high- and low-risk groups with significant survival differences. As the top candidates from this analysis, they were chosen to test whether ICT directly regulates these prognostic targets.

In this study, the expression patterns of FABP5 (red) and CYP2C9 (green) in HepG2 cells were evaluated using immunofluorescence double-labeling, with nuclei counterstained by DAPI (blue) (**Figure 11A**). Similarly, UCK2 (red) and CA9 (green) expression was examined. Fluorescence microscopy, combined with quantitative analysis using ImageJ (**Figure 11B**), was applied to estimate the impacts of different ICT concentrations on target protein expression. Relative to the control, FABP5 fluorescence intensity decreased in a DDM, with significant reductions observed at 15 μM and 30 μM (*p* < 0.01), whereas the 7.5 μM group exhibited a non-significant downward trend (*p* > 0.05). In contrast, CYP2C9 expression was significantly upregulated at 15 μM ICT (*p* < 0.01) and showed no significant change at 7.5 μM (*p* > 0.05). In the second detection group, UCK2 and CA9 expression levels were significantly downregulated in a DDM as ICT concentration increased (*p* < 0.05).

#### 3.2.4 The regulation of core target expression by ICT at the mRNA and protein levels

**Figure 12:**
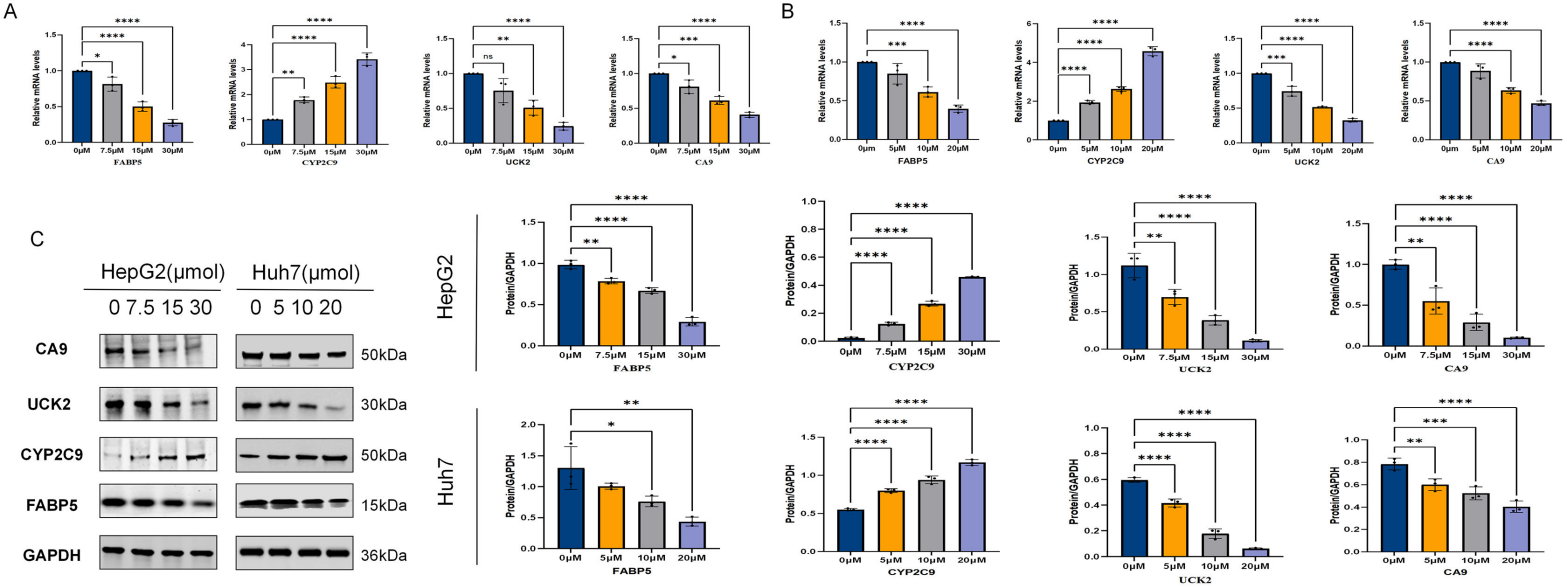
Effect of ICT on multi-target protein expression in HepG2 and Huh7 cells. **(A)** mRNA expression levels of relevant genes in HepG2 cells after 24-hour treatment with different concentrations of ICT, as determined by qPCR. **(B)** mRNA expression levels of relevant genes in Huh7 cells after 24-hour treatment with different concentrations of ICT, as determined by qPCR. **(C)** Western Blot analysis was used to determine the expression changes and quantification of CA9, UCK2, FABP5, and CYP2C9 proteins in HepG2 and Huh7 cells treated with different concentrations of ICT for 24 hours.(*p < 0.05, **p < 0.01, ***p < 0.001, ****p < 0.0001; Data are presented as the mean ± standard deviation (Mean ± SD) from at least three independent experiments (n = 3).

This study systematically evaluated the effects of ICT on the expression of key metabolism-related proteins—FABP5, UCK2, CA9, and CYP2C9—in HepG2 and Huh7 cells using qRT-PCR and Western blot analyses. As shown in **Figures 12A, B**, qRT-PCR results indicated that ICT dose-dependently suppressed the mRNA levels of FABP5, UCK2, and CA9, while gradually increasing CYP2C9 mRNA expression with rising ICT concentrations. Correspondingly, Western blot analysis (**Figure 12C**) revealed a marked reduction in the protein expression of FABP5, CA9, and UCK2, accompanied by a significant upregulation of CYP2C9 protein levels in response to increasing ICT concentrations. The overall concordance between mRNA and protein expression patterns suggests that ICT modulates these metabolism-related targets in a gene-specific and dose-dependent manner, exerting regulatory effects at both transcriptional and protein levels.

## 4 Discussion

HCC is among the most lethal malignant tumors worldwide and continues to pose a significant clinical challenge. Surgical resection remains the primary treatment but is limited to patients diagnosed at early stages, while pharmacological therapies, although capable of extending survival, are often constrained by drug resistance and adverse effects. Consequently, elucidating the pathogenesis of HCC and identifying more effective and safer therapeutic strategies have become critical research priorities. Advances in TCM research have provided growing evidence that plant-derived natural compounds exhibit potent anti-tumor activities, enhance therapeutic efficacy, reduce toxicity, and hold considerable promise for cancer prevention and treatment (34, 35). ICT, a major bioactive flavonoid extracted from the TCM herb Epimedium, has recently been demonstrated to inhibit HCC cell proliferation, induce apoptosis, modulate glycolytic metabolism, and promote cellular senescence (36, 37). Despite increasing interest in ICT’s therapeutic potential against HCC, its underlying molecular mechanisms remain incompletely understood. This study systematically investigates the molecular mechanisms through which ICT-related hub genes influence HCC prognosis and treatment, employing an integrated approach that combines network pharmacology, bioinformatics analysis, molecular docking, and *in vitro* experimental validation. Collectively, our findings provide both theoretical and experimental evidence providing a support to develop novel ICT-based therapies for HCC.

This study systematically elucidated the potential mechanisms underlying the antitumor effects of ICT against HCC by integrating network pharmacology, bioinformatics, and experimental validation. Through comprehensive multi-database analysis, 406 potential ICT targets were identified. Subsequent screening, which combined differential expression analysis, WGCNA, and intersection with HCC-associated genes, ultimately yielded 35 ICT-related DEGs. FEA indicated that these genes are primarily involved in critical signaling pathways such as cell cycle regulation, glycolysis, and drug metabolism. They participate in BPs related to cell cycle control and metabolic regulation, are associated with chromatin-related CCs, and are enriched in MFs such as organic acid binding and protein kinase activity. These findings suggest that ICT may exert its antitumor effects by modulating these key biological pathways. Among these pathways, cell cycle progression and glycolysis are well-established drivers of tumorigenesis, while protein kinases serve as central regulators of cellular energy metabolism, growth, proliferation, and survival (38, 39). Consistent with these predictions, CCK-8 and wound-healing assays demonstrated that ICT markedly inhibited the proliferation and migration of HepG2 and Huh7 cells in a dose- and TDM, thereby corroborating the bioinformatics-based findings.

In constructing a prognostic model, six candidate genes with prognostic significance were identified through the integration of univariate Cox regression and multiple MLAs. Among these, four core genes (UCK2, FABP5, CA9, and CYP2C9) were further refined using LASSO Cox regression and K-M survival analysis. The risk score model developed from these genes exhibited strong accuracy and stability in predicting 1-, 3-, and 5-year survival outcomes, underscoring its potential clinical applicability and prognostic value in HCC. Currently, sorafenib represents the standard first-line therapy for advanced HCC (40). Drug sensitivity analysis revealed significant correlations between the expression levels of these four genes and sorafenib sensitivity. Furthermore, the CIBERSORT algorithm is recognized as a gold-standard computational approach for assessing immune cell infiltration within the tumor microenvironment. Our analysis revealed that the risk score derived from the four-gene signature was positively correlated with the infiltration of M0 macrophages and regulatory T cells (Tregs)—both associated with pro-tumorigenic activity—and negatively correlated with the infiltration of M1 macrophages and monocytes, which are typically anti-tumorigenic. This consistent pattern of immune cell distribution suggests a close association between the risk model (and its constituent genes) and an immunosuppressive tumor microenvironment, thereby providing strong indirect evidence that ICT may modulate immune infiltration by targeting these genes. To further substantiate this hypothesis, molecular docking analysis was performed to investigate the interactions between ICT and the four core proteins. The results demonstrated that ICT exhibits strong binding affinities toward CA9, UCK2, FABP5, and CYP2C9, confirming potential direct molecular interactions with these targets. In addition, MD simulations further validated the stability and binding capacity of the ICT–protein complexes, reinforcing the proposed mechanism by which ICT may exert its therapeutic effects through modulation of these core targets.

Among the core genes identified in our study, CA9 is a direct downstream transcriptional target of HIF1α and has a pivotal role in regulating extracellular pH under hypoxic, acidic, and carcinogenic conditions. It is widely documented as a critical biomarker of tumor hypoxia (41), and hypoxia-induced drug resistance has been shown to compromise the efficacy of chemotherapy (42). Accumulating evidence indicates that CA9 expression is suggestively linked with poor clinical outcomes across various malignancies, including renal cell carcinoma, head and neck cancer, breast cancer, and gastric cancer (43). In HCC, CA9 upregulation promotes tumor progression by modulating cell proliferation, apoptosis, and epithelial-mesenchymal transition (EMT) (44, 45). In this study, analysis of the TCGA-LIHC cohort confirmed that CA9 expression is significantly upregulated in HCC tissues compared with normal liver tissues (**Figure 5I**). Within our constructed four-gene prognostic risk model, the coefficient for CA9 (0.0849) was markedly higher than those of the other genes, indicating that even minor fluctuations in CA9 expression exert a disproportionately large influence on the patient’s risk score. This finding identifies CA9 as the strongest risk factor within the model and the gene most closely associated with HCC prognosis. These observations are highly consistent with previous studies. For example, Huang WJ et al. reported that elevated CA9 expression serves as an independent predictor of poor prognosis in patients with resectable HCC, potentially by promoting EMT and enhancing tumor invasiveness (43). Similarly, another study demonstrated that CA9 overexpression, observed in approximately 61.8% (63/102) of HCC tumor samples, not only reflects a hypoxic and highly malignant tumor phenotype but also functions as a powerful, independent biomarker for unfavorable postoperative outcomes, including shortened survival and higher recurrence rates (46). In relation to ICT treatment, our *in vitro* experiments revealed that ICT significantly downregulates CA9 mRNA and protein expression in HepG2 and Huh7 cells in a DDM. Given CA9’s established role in regulating extracellular pH homeostasis, promoting EMT, and facilitating tumor invasiveness, its downregulation by ICT likely represents a key mechanism underlying ICT’s ability to inhibit HCC cell proliferation and migration, thereby contributing to its antitumor efficacy. Thus, the overexpression of CA9 not only reaffirms its prognostic value as a biomarker of poor outcomes but also supports its identification as a principal molecular target of ICT in HCC. In summary, considering CA9’s highest weighting in the prognostic model, its strong association with chemoresistance, and its central role in malignant progression—including hypoxia adaptation and EMT induction—CA9 can be regarded as the most critical and functionally significant gene within the identified risk signature.

Similarly, our data demonstrated that UCK2 and FABP5 are significantly overexpressed in HCC tissues, whereas CYP2C9 is markedly downregulated. Although CA9 exhibits the strongest relative importance, UCK2 and FABP5 also represent essential molecular drivers of HCC development and progression.UCK2 is a pyrimidine ribonucleotide kinase, catalyzing the phosphorylation of cytidine and uridine to produce uridine monophosphate and cytidine monophosphate, thereby participating in pyrimidine nucleotide metabolism (47). Aberrant overexpression of UCK2 has been linked with poor prognosis in multiple cancers, particularly in HCC (48), and our study further confirms that elevated UCK2 expression predicts adverse clinical outcomes, underscoring its potential as a prognostic biomarker. As a key enzyme involved in nucleotide synthesis during DNA replication, UCK2 overexpression may drive abnormal hepatoma cell proliferation. Moreover, increased UCK2 levels in HCC have been shown to promote tumor growth and metastasis by activating critical oncogenic signaling pathways, including STAT3, Wnt/β-catenin, and EGFR-AKT (49–51), whereas UCK2 knockdown significantly suppresses tumor cell proliferation (52). Inhibiting UCK2 directly disrupts the nucleotide “supply” for tumor cells, making it an attractive therapeutic target. Our results align with this: following ICT treatment, UCK2 expression was significantly suppressed, explaining ICT’s ability to inhibit HCC cell proliferation from the perspective of nucleotide metabolism. Targeting nucleotide metabolism is a classic anti-cancer strategy, thus establishing a solid theoretical foundation for UCK2 as a target of ICT.

FABP5 is a cytoplasmic transporter of oleic acid that participates in diverse biological processes, including cell proliferation, differentiation, and migration, all of which contribute to the development of multiple cancers (53). Clinical studies have illustrated that FABP5 overexpression is closely linked with poor clinical outcomes in HCC, colorectal cancer, and breast cancer, and it has emerged as a key oncogenic driver in liver tumorigenesis (54). Mechanistically, FABP5 improves HIF-1α activity by inhibiting its interaction with Factor Inhibiting HIF (FIH), thereby enhancing lipid accumulation and tumor cell proliferation (55). Furthermore, recent evidence indicates that FABP5 facilitates HCC progression through the CREB/miR-889-5p/KLF9 signaling pathway, underscoring its potential as a promising therapeutic and prognostic target in HCC (56). Consistent with previous reports, our study identified FABP5 as a pro-oncogenic factor in HCC. Treatment with ICT significantly downregulated FABP5 expression, suggesting that ICT may suppress the malignant phenotype of HCC cells by disrupting lipid metabolic pathways mediated by FABP5. Furthermore, molecular docking analysis revealed a low binding energy between ICT and FABP5, indicating a strong and stable interaction that may underlie ICT’s inhibitory effect on FABP5 activity.

Additionally, CYP2C9 belongs to the cytochrome P450 2C (CYP2C) family and is critically CYP2C9, an enzyme involved in the metabolism of numerous carcinogens and pharmaceutical agents, exhibits significantly reduced expression in HCC (57). Time-series transcriptomic analyses in mouse models have demonstrated that CYP2C9 plays a critical regulatory role in HCC development by suppressing hepatocyte proliferation induced by liver injury through inhibition of the NF-κB signaling pathway. Furthermore, human CYP2C9 expression in mice has been positively associated with improved OS in HCC patients, illuminating its potential protective role in HCC (58). This finding makes ICT’s regulatory effect on CYP2C9 particularly distinctive and significant. In contrast to its inhibitory effects on other genes, our experiments demonstrated that ICT treatment markedly upregulates CYP2C9 protein expression in both HepG2 and Huh7 cells. This upregulation may help restore partial differentiation functions in hepatocytes or indirectly suppress tumor growth. Thus, the combination of basal CYP2C9 underexpression in HCC and ICT’s specific upregulatory effect provides a compelling rationale for identifying CYP2C9 as a key therapeutic target of ICT. In summary, ICT appears to function through a dual regulatory strategy: “suppressing malignancy” by downregulating pro-oncogenic genes (CA9, UCK2, and FABP5), and “reinforcing physiological function” by upregulating the potential tumor suppressor CYP2C9. This bidirectional, multi-target regulatory mechanism underscores ICT’s therapeutic advantage in achieving comprehensive modulation of tumor biology. We propose that ICT exerts its multi-target anti-HCC activity through synergistic mechanisms—inhibiting CA9 (microenvironment regulation), UCK2 (nucleotide metabolism), and FABP5 (lipid metabolism), while enhancing CYP2C9 expression, which collectively contribute to its potent antitumor efficacy.

In conclusion, this study integrates computational biology and experimental validation to demonstrate that ICT potentially inhibits tumor progression and holds prognostic significance in HCC by modulating a novel network of core targets—CA9, UCK2, FABP5, and CYP2C9—thereby influencing key oncogenic pathways, including the cell cycle and metabolic regulation. It is noteworthy that ICT, as a multi-target agent, exerts its anti-HCC effects through complex network-based mechanisms. Previous studies have reported alternative targets and mechanisms for ICT. For instance, Mo D et al. revealed that ICT directly binds to and inhibits IKKα, thereby downregulating tumor cell PD-L1 expression via the NF-κB signaling pathway, which alleviates T-cell suppression and enhances anti-tumor immune responses (59). Another network pharmacology investigation predicted that ICT may interact with additional potential targets (60). The differences between these findings and our results likely reflect distinct research emphases and methodological approaches. Specifically, our study focused on identifying ICT targets most closely associated with HCC prognosis through systematic bioinformatic screening and *in vitro* validation, whereas other studies have emphasized specific BPs such as immune regulation or have employed broader predictive frameworks. Importantly, these findings are not mutually exclusive but instead represent complementary aspects of ICT’s multi-dimensional anti-HCC activity. Collectively, they depict ICT as acting on both tumor-intrinsic pathways (e.g., metabolism and proliferation, as emphasized in this study) and the tumor microenvironment (e.g., immunomodulation), embodying the multi-component, multi-target, and multi-pathway therapeutic paradigm characteristic of TCM. Future studies—particularly those employing integrated analyses in immunocompetent animal models—are warranted to further elucidate the systemic and synergistic mechanisms underlying ICT’s anti-HCC efficacy and to facilitate its translation into clinical application.

Furthermore, although this study identified potential ICT targets in HCC through integrated analytical approaches and provided *in vitro* validation, and although we explored the relationship between these gene expression patterns and immune cell infiltration at the bioinformatic level, several limitations should be acknowledged. Most notably, *in vivo* experimental data are currently lacking to confirm whether ICT can similarly modulate the expression of CA9, UCK2, FABP5, and CYP2C9, as well as remodel the tumor immune microenvironment in living organisms. Future studies will employ murine HCC models to directly validate these regulatory effects and mechanistic insights. Despite these limitations, the present work offers valuable candidate targets and establishes a clear scientific foundation for subsequent *in vivo* and clinical investigations.

## 5 Conclusion

This study systematically integrated network pharmacology, molecular docking, bioinformatics analysis, and *in vitro* experiments to elucidate the molecular mechanisms underlying ICT’s therapeutic effects on HCC. Our findings demonstrate that ICT significantly inhibits the proliferation and migration of HCC cells in both the DDM and TDM, potentially through modulation of key signaling pathways, including cell cycle regulation and metabolic reprogramming. Moreover, ICT exerts its anti-HCC effects, at least in part, by downregulating the expression of CA9, UCK2, and FABP5 while upregulating CYP2C9, thereby validating these four genes as robust prognostic biomarkers and potential therapeutic targets. Collectively, these results provide a novel theoretical foundation for targeted HCC therapy and highlight ICT as a promising therapeutic agent with substantial potential in HCC treatment.

## Data Availability

The original contributions presented in the study are included in the article/**Supplementary Material**. Further inquiries can be directed to the corresponding authors.
