# Supplementary material for: Identification and validation of icaritin-associated prognostic genes in hepatocellular carcinoma through network pharmacology, bioinformatics analysis, and cellular experiments
Source: Front Immunol. 2025 Nov 20;16:1693028. doi: 10.3389/fimmu.2025.1693028 (PMC12675460; doi:10.3389/fimmu.2025.1693028)
Supplement: Supplementary file 1 [file Table1.docx]

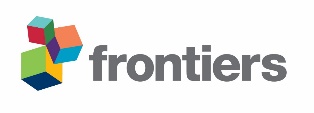
Supplementary Material

Supplementary Table 1: 35 ICT-related differentially expressed genes in HCC

| No. | Gene name | No. | Gene name | No. | Gene name |
| --- | --- | --- | --- | --- | --- |
| 1 | CHEK1 | 13 | CYP2C9 | 25 | CYP1A2 |
| 2 | KIF11 | 14 | ADH1C | 25 | CYP3A4 |
| 3 | TTR | 15 | FABP5 | 27 | DKK1 |
| 4 | CCNA2 | 16 | ALDH2 | 28 | PKM |
| 5 | ANG | 17 | SERPINA1 | 29 | WNT3A |
| 6 | SRC | 18 | FABP6 | 30 | CDK1 |
| 7 | TYMS | 19 | RBP4 | 31 | MELK |
| 8 | AURKA | 20 | UCK2 | 32 | TOP2A |
| 9 | C1R | 21 | DTYMK | 33 | PLK1 |
| 10 | AKR1C3 | 22 | ARG2 | 34 | NEK2 |
| 11 | PCK1 | 24 | TK1 | 35 | CHEK2 |
| 12 | CA9 | 24 | ALPL |  |  |
